# On Premature Puberty

**Published:** 1810-11

**Authors:** 


*To the Editors of the Medical and Physical Journal.*

### *On Premature Puberty.*

GENTLEMEN,

IN the Paper giving an "Account of Philip Howorth, in whom signs of puberty appeared at a very early age," published by Mr. White, in the *Medico-Chirurgical Transactions*, Vol. 1. we are referred to one or two instances of a similar nature, in other boys; it has been asked whether such instances of premature puberty have ever occurred in the female sex, but I do not remember to have seen or heard any such referred to on this occasion. The subject, however, is curious, and may be worthy of investigation. I beg leave, therefore, to mention a publication, not generally known, in which a case of this kind, in a female, is recorded. My information is gained from Monsieur Sue's curious farrago, which he has entitled "Essais Historiques, litteraires, et critiques sur l'Art des Accouchemens," 2 tom. Paris, 1779.—Speaking of Mons. Simon's "Recherches sur l'operation césarienne," Sue says, he was likewise the Editor of a Collection, in 4 vols. 12mo. of various Tracts on Surgery, Anatomy, and the Practice of Medicine, chiefly extracted from foreign publications, at Paris, in the year 1761, in which are several papers relating to Midwifery. At the beginning of the second volume, is published a letter from Mons. Schmith, giving an account of a girl, who, at the age of eight years and ten months, was delivered of a dead child, at the full

 term term, having both its hair and nails. This young mother had been *regular* ever since the age of two years, and the parts of generation resembled those of young women of seventeen or eighteen years of age.—Essais, &c. par M. Sue le jeune, tom. 2. p. 344.

Should any of your Correspondents be in possession of this publication of Simon's, they will probably favour you with more particulars of this case.

The curious and authentic history of Philip Howorth, would almost tempt one to give credit to some of the extraordinary relations of the older writers, which have hitherto been considered as completely fabulous. I take the liberty of subjoining some of these, extracted from "*An Essay on the Possibility and Probability of a Child's being born alive, and live, (living) in the latter end of the fifth solar, or in the beginning of the sixth lunar month.*"—*By David Dickson, M. D. C. R. M. E. S. Edinburgh* 1712.

"*St. Jerome* in his 132d Epistle directed to *Bitalis*, makes mention of a boy, of ten years of age, impregnating his nurse, and for the verity of the fact, he takes God to witness, he did not lie." That is to say, he did not lie in reporting what he had heard.

"Albertus Magnus tells, that *he knew* a girl with child in the ninth year of her age, who was delivered of the same in the tenth."

"*Joseph Scaliger* writes in his Chronological Epistle to *Gomarus*, of a girl of ten years of age, who bore a son to her cousin-german who was not full twelve; a matter of fact known to all the inhabitants of Gascony."

"*Jacobus a Partibus*, in his Commentaries upon Avicenna, Tract. i. Cap. 12, asserts that *he saw* a girl of Tournay, of nine years of age, who was a mother: so that it is possible she might have conceived when she was only eight."

Several other equally extraordinary stories of this kind are collected together by Dr. Dickson, but I think these are sufficient; I shall only add one more, viz. that "*Busbequius* in his description of *Colchis*, an Asiatic country, now called *Mengrelia*, tells us, that the girls there are for the most part mothers in the tenth year of their age; but if this seems not only wonderful but incredible, then they are ready to shew their children, who scarcely exceed the bigness of a frog, to any who desire a sight of them."

Had Dr. Dickson confined himself to the former instances, he would at least have passed for a grave author and diligent compiler, but there is something so ludicrous in the idea of a number of children being shewn altogether, (he must mean alive) not bigger than frogs, as cannot fail to excite laughter.

*Oct.* 15, 1810. M.

